# NAD^+^ in Alzheimer’s Disease: Molecular Mechanisms and Systematic Therapeutic Evidence Obtained *in vivo*

**DOI:** 10.3389/fcell.2021.668491

**Published:** 2021-08-03

**Authors:** Xinshi Wang, Hai-Jun He, Xi Xiong, Shuoting Zhou, Wen-Wen Wang, Liang Feng, Ruiyu Han, Cheng-Long Xie

**Affiliations:** ^1^Department of Neurology, The First Affiliated Hospital of Wenzhou Medical University, Wenzhou, China; ^2^The Center of Traditional Chinese Medicine, The Second Affiliated Hospital and Yuying Children’s Hospital of Wenzhou Medical University, Wenzhou, China; ^3^National Health Commission (NHC) Key Laboratory of Family Planning and Healthy, Hebei Key Laboratory of Reproductive Medicine, Hebei Research Institute for Family Planning Science and Technology, Shijiazhuang, China; ^4^Key Laboratory of Alzheimer’s Disease of Zhejiang Province, Wenzhou, China; ^5^Institute of Aging, Wenzhou Medical University, Wenzhou, China; ^6^Oujiang Laboratory, Wenzhou, China

**Keywords:** NAD^+^, NAD^+^ precursors, Alzheimer’s disease, aging, molecular mechanisms

## Abstract

Mitochondria in neurons generate adenosine triphosphate (ATP) to provide the necessary energy required for constant activity. Nicotinamide adenine dinucleotide (NAD^+^) is a vital intermediate metabolite involved in cellular bioenergetics, ATP production, mitochondrial homeostasis, and adaptive stress responses. Exploration of the biological functions of NAD^+^ has been gaining momentum, providing many crucial insights into the pathophysiology of age-associated functional decline and diseases, such as Alzheimer’s disease (AD). Here, we systematically review the key roles of NAD^+^ precursors and related metabolites in AD models and show how NAD^+^ affects the pathological hallmarks of AD and the potential mechanisms of action. Advances in understanding the molecular roles of NAD^+^-based neuronal resilience will result in novel approaches for the treatment of AD and set the stage for determining whether the results of exciting preclinical trials can be translated into the clinic to improve AD patients’ phenotypes.

## Background

Nicotinamide (NAM) adenine dinucleotide (NAD^+^) is well known as a vital coenzyme that enables rewire metabolism process in cellular bioenergetics and maintain mitochondrial homeostasis and adaptive stress responses to oxidative damage (Yaku et al., 2018). It is a crucial cofactor for more than 500 enzymatic reactions and plays a pivotal role in the management of almost all major biological metabolism in the cytosol, nucleus, and mitochondria that determine cellular health. Diverse lines of research have indicated that a decline in NAD^+^ level is a fundamental trait of aging that may predispose individuals to a large spectrum of chronic diseases (Canto et al., 2015). Specifically, recent discoveries have indicated a steady age-dependent decline in NAD^+^ levels, resulting in an altered metabolic rate and increased disease susceptibility. Moreover, NAD^+^ depletion has been closely connected with multiple hallmarks of aging (Fang et al., 2017). Restoration of NAD^+^ is important for disparate cells with high energy demands; and physiological (e.g., exercise and food intake) and pharmacological interventions (e.g., NAD^+^ precursor supplementation) bolstering cellular NAD^+^ levels in old or diseased animals might slow the processes of aging, promote health and extend life span, conferring benefit for resisting not only one disease but also many diseases, thereby facilitating health (Yoshino et al., 2018).

## NAD^+^ Biosynthesis and Metabolic Pathways

**FIGURE 1:**
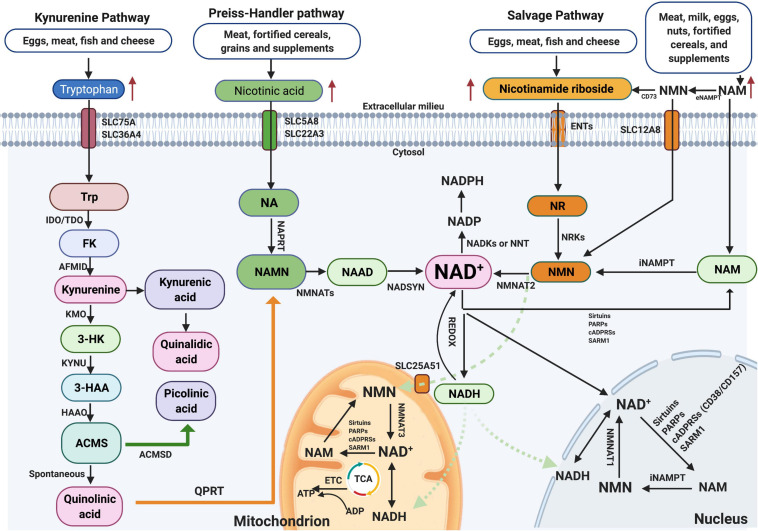
NAD^+^ biosynthesis pathway and subcellular equilibrium: NAD^+^ is synthesized via three major pathways, including *de novo* biosynthesis (also called the kynurenine pathway), the Preiss–Handler pathway, and the salvage pathway. The kynurenine pathway starts with the catabolism of the amino acid Trp that is converted to FK, which is further converted to kynurenine. Kynurenine can be converted to KA and finally QA. In addition, kynurenine can be converted to 3-HK by KMO and further to 3-HAA by KYNU. The next step is performed by 3HAAO to produce ACMS and then converts to QA, which further formulates to NAMN by QPRT and finally to NAD^+^. NAD^+^-consuming enzymes include sirtuins, PARPs, cADPRSs, and SARM1. The equilibrium of NAD^+^ is a balance of synthesis, consumption, and recycling in various subcellular compartments including the cytosol, the nucleus, and the mitochondria. NAD^+^, nicotinamide adenine dinucleotide; KA, kynurenic acid; 3-HK, 3-hydroxykynurenine.

NAD^+^ is one of the most consequential molecules in the human body at average level of approximately 3.0 g. NAD^+^ is synthesized mainly via three pathways, including *de novo* biosynthesis pathway (also called the kynurenine pathway), the Preiss–Handler pathway, and the salvage pathway (Figure 1), not only in the cytosol but also within major organelles (Anderson et al., 2003). The kynurenine pathway is initiated by the catabolism of tryptophan, which is converted via several steps to the kynurenine, which can generate NAD^+^, quinaldic acid (QA), or picolinic acid (PA) (Vecsei et al., 2013). The Preiss–Handler pathway is based on nicotinic acid (NA) via the intermediates NA mononucleotide (NAMN) and NA adenine dinucleotide (NAAD). Important enzymes in this pathway are NAM mononucleotide (NMN) adenylyl transferases (NMNATs), including NMNAT1–3 (Ali et al., 2013). In the salvage pathway, NAD^+^ is synthesized from NAM through the intermediate NMN by NAM phosphoribosyl transferase (NAMPT) (Yang et al., 2007), which is the key step in efficient NAD^+^ synthesis. Additionally, to maintain NAD^+^ at stable levels, most NAD^+^ is reused by the salvage pathway, not the *de novo* or Preiss–Handler pathway. Detailed information on these pathways is summarized in Figure 1 and has been recently reviewed elsewhere (Verdin, 2015; Lautrup et al., 2019). Interestingly, the kynurenine pathway also produces a lot of metabolites, such as 3-hydroxykynurenine (3-HK) and the excitotoxin quinolinic acid. Due to the opposite roles of the neuroactive metabolites of this pathway in neurodegenerative diseases, 3-HK and quinolinic acid show neurotoxicity, while kynurenic acid (KA) is neuroprotective (Tan et al., 2012), and therefore, targeting individual metabolites may be particularly beneficial for developing treatments for Alzheimer’s disease (AD). Regarding NAD^+^-consuming enzymes, NAD^+^ is a cofactor for at least three kinds of enzymes that catabolize NAD^+^ to NAM, including SIRTs, cADPRSs, and PARPs. Recently, NAD^+^ has attracted substantial eyes as a target in the aging research field, partially with respect to the sirtuin (SIRT) family (SIRT1–7). In this context, SIRTs constitute a group of NAD^+^-dependent deacetylases and ADP-ribosyltransferases that deacetylate or deacylate target proteins, affecting inflammation, neuronal function, energy metabolism, stress resistance, etc. (Gertler and Cohen, 2013; Bonkowski and Sinclair, 2016).

Notably, the presence of NMNATs and NAMPT in the cytosol, mitochondria, and/or nucleus indicates that salvaging of NAD^+^ from NMN and NAM is evident in these compartments (Figure 1). NAD^+^-consuming enzymes, such as SIRTs, PARPs, cADPRSs (CD38/CD157), or SARM1, are also present in these compartments, promoting the constant equilibrating cycle of NAD^+^ biosynthesis, degradation, and recycling in these regions. Among these enzymes, SIRTs, PARPs, and CD38 are three main kinds of NAD^+^-consuming enzymes, and they compete for bioavailable NAD^+^. Hence, hyperactivity of one enzyme might inhibit the activity of the others, and vice versa. SIRTs constitute a group of NAD^+^-dependent deacetylases and ADP-ribosyltransferases that deacetylate or deacylate target proteins (Bonkowski and Sinclair, 2016). PARPs are primary members of the ADP-ribosyltransferase family, with 17 different enzymes in mammals, but among these enzymes, only four (PARP1, PARP2, PARP5a, and PARP5b) are capable of adding multiple ADP-ribose units to substrates or undertaking PARylation (Rouleau et al., 2010). CD38 plays a role as a glycohydrolase or NADase, hydrolyzing NAD^+^ to generate NAM and cADPR as a side products, while also hydrolyzing cADPR to generate ADPR; this reaction generates NAM, which is rapidly regenerated to NAD^+^ via the salvage pathway (Quarona et al., 2013).

Moreover, p53 is a known target of SIRT1. Given the vital role of p53 in the apoptotic response, numerous papers have reported the regulation of p53 via SIRT1. As a transcription factor, p53 induces apoptosis and is inhibited by SIRT1 deacetylation at several cellular locations, and SIRT1-deficient cells possess hyperacetylated p53 (Luna et al., 2013). In contrast, p53 can inhibit SIRT1 expression when nutrients are abundant through p53-binding sites on the SIRT1 promoter (Park et al., 2021). In the past decade, emerging evidence has indicated that the function of p53 is directly modulated by PARP-1 (Elkholi and Chipuk, 2014). An interaction between p53 and PARP-1 has also been reported *in vitro* undergoing apoptosis in response to DNA damage. Furthermore, p53 transcriptional activity is mediated by PARP-1, as well as enhanced p53-dependent apoptosis (Akpolat et al., 2020).

Nonetheless, different isoforms of NMNATs reside in different cellular compartments (e.g., NMNAT1 in the nucleus, NMNAT2 in the cytosol, and NMNAT3 in the mitochondria), which indicates that NAD^+^ salvage is regulated based on subcellular compartment-specific metabolic needs and behaves independently (Yang et al., 2007). Further, as no NRTs exist in the mitochondrial pool, the increase in mitochondrial NAD^+^ levels after NR treatment observed in cultured cells was likely due to the conversion of NR to NMN in the cytosol, and NMN traverses the mitochondrial membrane to generate NAD^+^ by NMNATs (Canto et al., 2012). Moreover, several studies have reported that both NMN and NAD^+^ can be transported into mitochondria via unclear mechanisms (Davila et al., 2018). A recent paper has found that Slc12a8 knockdown abrogates the uptake of NMN and decreases NAD^+^ levels both *in vitro* and *in vivo*, suggesting that SLC2A8 is a specific NMN transporter in the mouse small intestine (Grozio et al., 2019). Moreover, lack of SLC25A51 has been shown to decrease mitochondrial NAD^+^ content and block the uptake of NAD^+^ by isolated mitochondria, indicating that SLC25A51 is a mammalian NAD^+^ transporter in mitochondria (Luongo et al., 2020). NAM is an uncharged molecule that can freely diffuse across the plasma and mitochondrial membranes. Furthermore, some NAD^+^ transporters, such as SLC5A8 (which takes up NA), SLC75A (which takes up tryptophan), and SLC25A17 (which takes up NAD^+^), localize in the cell and peroxisome membranes (Figure 1; Agrimi et al., 2012).

## NAD^+^-Boosting Molecules

**FIGURE 2:**
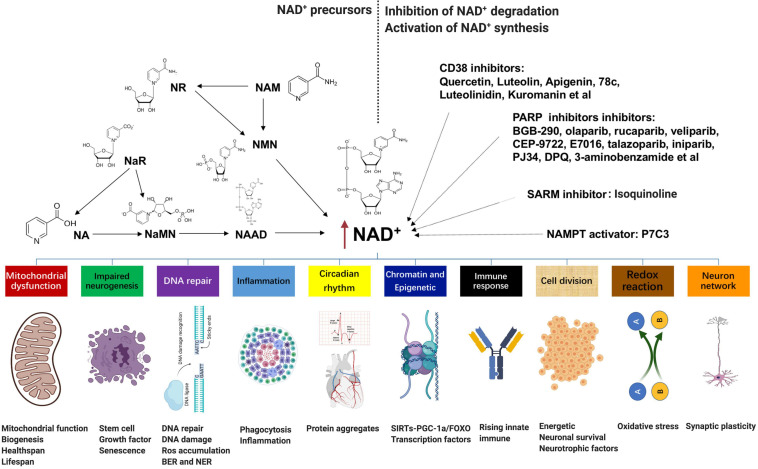
NAD^+^-boosting molecules and crosstalk with several hallmarks of aging: supplementation with NAD^+^ precursors (mainly NR, NMN, NAM, and NA), activation of NAD^+^ biosynthetic enzymes (e.g., NAMPT activator P7C3), and inhibition of NAD^+^ degradation (CD38 inhibitors quercetin, luteolin, apigenin, 78c, luteolinidin, and kuromanin; PARP inhibitors BGB-290, olaparib, rucaparib, veliparib, CEP-9722, E7016, talazoparib, iniparib, PJ34, DPQ, and 3-aminobenzamide; and SARM1 inhibitor isoquinoline) are three main approaches to increase NAD^+^ levels. Fluctuations in NAD^+^ impact several hallmarks of aging, such as mitochondrial dysfunction, neurogenesis, DNA repair, immune response and inflammation, compromised autophagy, chromatin and epigenetics, cell division, redox reaction, and neuron network. NAD^+^, nicotinamide adenine dinucleotide; NMN, nicotinamide mononucleotide; NAM, nicotinamide; NA, nicotinic acid.

Numerous pathophysiological conditions display obvious decline NAD^+^ level in tissue, such as aging, obesity, and neurodegeneration. Supplementation with NAD^+^ precursors (mainly NR, NMN, NAM, and NA), activation of NAD^+^ biosynthetic enzymes (e.g., NAMPT activator P7C3), and inhibition of NAD^+^ degradation (CD38 inhibitors, PARP inhibitors, and SARM1 inhibitors) are three main approaches to increase NAD^+^ levels (Figure 2; Kang et al., 2020; Luongo et al., 2020). For the NAD^+^ precursors, NR, NAM, or NMN can increase NAD^+^ content (Canto et al., 2015). Among them, NR and NMN are the most common and effective agents to increase the NAD^+^ level because they are soluble and orally bioavailable (Conze et al., 2016; Trammell et al., 2016; Yoshino et al., 2018). In rodents, NR is much more efficient in increasing NAD^+^ contents than NA and NAM, potentially because of increased uptake. The ability of NA to enhance the NAD^+^ level has been shown in several studies; however, its application is confined due to unpleasant side effects (Rolfe, 2014). Besides, NaR, NaMN, and NAAD are all potential agents to boost the NAD^+^ contents.

An alternative method to enhance NAD^+^ is directly overexpressing or provoking NAD^+^ biosynthetic enzymes. For instance, NAMPT is the primary rate-limiting enzyme in the process of NAM to NMN, which is also declined with age and several disease stress. However, most enzymes are currently under investigation except that P7C3 was found to possess relatively weak NAMPT activity *in vitro* (Wang et al., 2014). In addition, epigallocatechin gallate from the green tea has been shown to activate NMNAT2 and NMNAT3 by more than 100 and 43%, respectively (Berger et al., 2005). In terms of the inhibition of NAD^+^ degradation, either pharmacological or genetic inhibition of PARP-1 or CD38 is adequate to add tissue NAD^+^ (Pirinen et al., 2014). Overexpression of CD38 *in vitro* leads to about 35% decline in NAD^+^ (Hu et al., 2014); in contrast, quercetin, luteolin, apigenin, 78c, luteolinidin, and kuromanin, which appear to inhibit CD38, are also potentially efficient to increase NAD^+^ (Kellenberger et al., 2011). Furthermore, PARP can be inhibited by BGB-290, CEP-9722, E7016, talazoparib, olaparib, rucaparib, veliparib, iniparib, PJ34, DPQ, and 3-aminobenzamide (Brown et al., 2016). All of these molecules are potential agents to raise the NAD^+^ level, but most of them are still under research, and the translational potential of the latter two approaches may be lower than that of the first strategy.

## NAD^+^ and the Hallmarks of Alzheimer’s Disease

**FIGURE 3:**
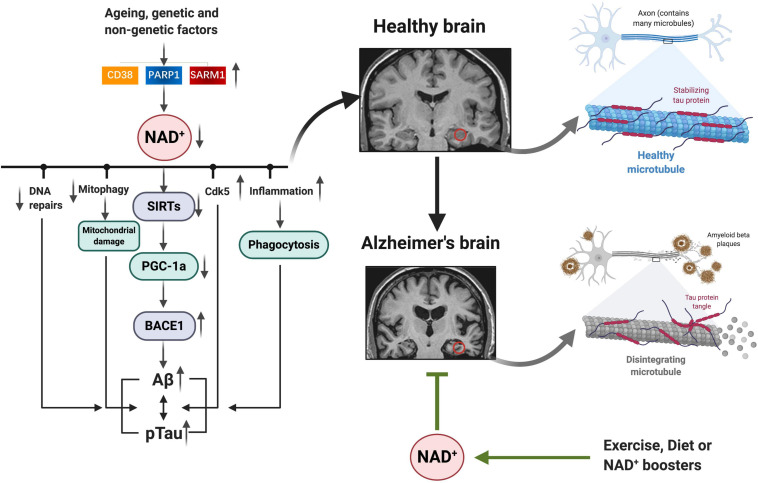
NAD^+^ depletion involved in AD pathophysiology hallmarks: as we age, or under some genetic and non-genetic factors, increased NAD^+^ consumption (CD38, PARP1, and SARM1) drives NAD^+^ depletion, leading to impaired DNA repair, mitochondrial homeostasis, and neuronal function, as well as inflammation, all of which may exacerbate the disease progression. Emerging evidence indicates a vital role for NAD^+^ depletion and impairment of NAD^+^-dependent metabolism pathways involved in AD pathophysiology hallmarks (Aβ plaques, hyperphosphorylation of tau, and mitochondrial dysfunction); and NAD^+^ boosting by exercise, diet, or pharmacological boosters can improve cognitive deficits in AD models by suppressing these pathologies. NAD^+^, nicotinamide adenine dinucleotide; AD, Alzheimer’s disease.

Fluctuations in NAD^+^ impact several hallmarks of aging, such as mitochondrial dysfunction, neurogenesis, immune response, DNA repair, compromised autophagy, chromatin and epigenetic, cell division, redox reaction, and neuron network (Figure 2; Fang et al., 2017; Rajman et al., 2018). AD is the most prevalent type of dementia, but there are still no effective treatments that affect the progression of AD (Livingston et al., 2017). The pathology hallmarks of AD are characterized by the accumulation of amyloid-β (Aβ) plaques, as well as tau neurofibrillary tangles (NFTs) and mitochondrial dysfunction in the brain. Emerging evidence indicates a vital role for depletion of NAD^+^ and impairment of NAD^+^-dependent metabolism pathways involved in AD pathophysiological hallmarks, and NAD^+^ supplementation can improve cognitive deficits in cross-species AD models (Figure 3).

### NAD^+^ Crosstalk With Amyloid-β

Aβ accumulates in the brain cortex and hippocampus, mainly due to defective clearance of the peptide (Hardy and Higgins, 1992). This cascade hypothesis showed that Aβ accumulation in the brain actuates tau phosphorylation, NFT formation, neuron death, and cognitive deficits. Three months of NR (250 mg/kg/day) treatment has been documented to attenuate Aβ toxicity resulting from reduced Aβ production via PGC-1α-mediated BACE1 degradation in the cortex and hippocampus in Tg2576 AD mouse model (Gong et al., 2013). In APP/PS1 mice, treatment with NR could decrease the chronic neuroinflammation and the number or total area of Aβ plaques in the cortex (Xie et al., 2019). In addition, treatment of NMN results in apparent reduction of Aβ levels and also Aβ oligomers by inhibiting JNK activation (Yao et al., 2017). Knockdown of CD38 in APP/PS1 mice reduced Aβ plaques and soluble Aβ levels, as well as improved the spatial learning and memory (Blacher et al., 2015). Accordingly, in 3xTg-AD model, brain NAD^+^ redox-dependent reaction depletion and metabolic dysfunction have been demonstrated, which may contribute to decreased energetic capacity (Dong and Brewer, 2019). The metabolomics profiling of hippocampal was carried out in control and 5xFAD mice at 6, 8, and 12 months, and the NAM level was significantly lower in 5xFAD mice than in wild-type (WT) mice (Kim et al., 2020).

### NAD^+^ Crosstalk With Tau

In AD, tau exists as monomers; oligomeric, paired helical filaments (PHFs); and straight filaments (Meraz-Rios et al., 2010). Hyperphosphorylation of tau (p-tau) is one of the core events in the progress of AD, and the extent of phosphorylation reveals abnormal activity of both upstream protein kinases and phosphatases. Of note, p-tau is also an essential element for the formation of NFTs, and inhibition of p-tau by NAD^+^ boosting has been reported in several studies. Administered NAM reduced p-tau (Thr231) and improved cognitive performance in 3xTgAD mice but did not decrease p-tau in late-stage mice (Green et al., 2008). In a worm model of tauopathy with pan-neuronal expression of the tau fragment (BR5270), NMN 5 mM for this *Caenorhabditis elegans* tauopathy model induced memory relieve, which was dependent on DCT-1 function (Fang et al., 2019). Moreover, NAM-loaded functionalized solid lipid nanoparticles (SLNs), a sustained/controlled delivery system, could ameliorate the cognition impairment and preserve the neuronal cells in AD rat model (Vakilinezhad et al., 2018). In the 3x TgAD and 3x TgAD/Polβ^±^ models, NR treatment for 6 months showed significantly decreased p-tau (Thr231) levels in the hippocampus and cortex using immunofluorescence and immunoblot analysis (Hou et al., 2018).

### NAD^+^ Crosstalk With Mitochondrial Dysfunction

Mitochondria are an important cell organelle referred to as the “powerhouses” of cells and produces adenosine triphosphate (ATP) by oxidative phosphorylation to support neuron activities. Recent studies reported that neurons affected in AD brain accumulate dysfunctional or damaged mitochondria in part due to mitophagy deficit (Cummins et al., 2019). Notably, NAD^+^ is dedicated to keeping mitochondrial fitness and improving mitochondrial biogenesis, mitochondrial unfolded protein response (UPR^*mt*^), and mitophagy (Katsyuba and Auwerx, 2017). NAD^+^ influences neuronal homeostasis and survival by maintaining the balance between mitophagy and mitochondrial biogenesis via the NAD^+^–SIRT1–PGC-1a pathway or the DAF-16/FOXO3 pathway (Fang et al., 2016). Supplementation with NMN could reduce the impaired mitochondria and improve the mitochondria quality control in human AD patient induced pluripotent stem cell (iPSC)-derived neurons and in AD mouse and *C. elegans* models, and this in turn forestalls synaptic dysfunction and improves memory and learning (Fang et al., 2019).

## NAD^+^ Precursors as a Potential Therapeutic Intervention for Alzheimer’s Disease Models

Pretreatment of neurons with NAD^+^ precursors protects against axonal degeneration following axotomy or even noise-induced hearing loss in mice, presenting a neuroprotection effect (Brown et al., 2014). Moreover, induction of NAD^+^ levels after calorie restriction attenuated Aβ accumulation (Qin et al., 2006). It is of interest that transgenic AD mice ameliorate memory loss by NR treatment (Gong et al., 2013). Restoration of neuronal mitophagy by NMN could reduce Aβ_1__–__42_ and Aβ_1__–__40_ levels and cognitive decline in both *C. elegans* and AD mouse model through microglial phagocytosis and suppression of neuroinflammation (Fang et al., 2019). We should notice that NAD^+^ precursors, such as NAM, NMN, and NR, may be an intriguing way to stop AD progression.

## Systematic Review of Preclinical Trials of NAD^+^ Precursors for Alzheimer’s Disease Models

We systematically searched PubMed, Google Scholar, and the Cochrane Library from inception to May 30, 2020. Finally, we identified 14 eligible preclinical trials of NAD^+^ precursors for AD models (Green et al., 2008; Gong et al., 2013; Liu et al., 2013; Bayrakdar et al., 2014a,b; Long et al., 2015; Wang et al., 2016; Kim and Yang, 2017; Yao et al., 2017; Hou et al., 2018; Vakilinezhad et al., 2018; Fang et al., 2019; Xie et al., 2019; Kim et al., 2020). The main information of the included preclinical trials testing the effect of NAD^+^ precursors on AD models is summarized in Table 1. Meanwhile, there were no eligible preclinical trials of NA for AD models.

**TABLE 1:** Mainly features of the included preclinical studies testing the effect of NAD^+^ precursors on AD models.

**References**	**AD models**	**Intervention regimen**	**Results**
**NAM vs. placebo**			
Kim et al. (2020)	5xFAD	Treatment with 10 mM of NAM for 24 h in hippocampal primary neurons	1. Hippocampal metabolomics at different disease progression stages 2. NAM rescues the spine deficits in hippocampal primary neurons
Vakilinezhad et al. (2018)	Streptozotocin (STZ)-induced AD rats model	NAM-loaded functionalized solid lipid nanoparticles (SLNs)	1. Body weight and blood glucose levels 2. Spatial learning and memory test—Morris water maze 3. Histopathology and biochemical studies (p-tau)
Kim and Yang (2017)	Aβ-injected ICR mice	NA 20 mg/kg/day and NAM 200 mg/kg/day pretreatment for 7 days	1. Aβ_1__–__42_ tail vein injection increased brain Aβ_1__–__42_ levels 2. NA had no effect on the expression of APP and PS1. 3. NAM significantly reduced gene expression of APP and PS1 in brain tissues
Bayrakdar et al. (2014a)	Aβ-injected rat AD model	NAM (100 and 500 mg/kg) was introduced intraperitoneally (i.p.) for 7 days	1. NA ameliorates Aβ_1__–__42_-induced oxidative stress and disruption of antioxidant defense 2. NA reduces Aβ_1__–__42_ mRNA and protein levels of PARP-1 and NF-κB 3. NA inhibits apoptosis induced by Aβ_1__–__42_
Bayrakdar et al. (2014b)	Aβ-injected rat AD model	NAM (100 and 500 mg/kg) i.p. for 7 days	1. Protective effects of NAM against Aβ_1__–__42_-induced mitochondrial dysfunction, protein oxidation, and lipid oxidation 2. Protective effects of 3-AB and NAM against Aβ_1__–__42_-induced ROS production
Liu et al. (2013)	3xTgAD	Administered NAM in the drinking water (40 mg/kg/day) for 8 months	1. NAM protects neurons from oxidative damage and Aβ toxicity 2. NAM ameliorates cognitive decline and neuropathology in 3xTgAD mice 3. NAM improves mitochondrial function and autophagy-lysosome procession 4. NAM increases p-Akt, MAPK/ERK1/2, and CREB signaling pathways
Green et al. (2008)	3xTgAD	Administered NAM in the drinking water (200 mg/kg/day) for 4 months	1. NAM prevents cognitive deficits in AD mouse model 2. NAM does not affect Aβ pathology 3. NAM selectively reduces Thr231 phosphorylated tau
**NMN vs. placebo**			
Fang et al. (2019)	1. Tau and Aβ *C. elegans* 2. APP/PS1	NMN 5 mM for *C. elegans*	1. NMN ameliorates cognitive decline in *C. elegans* models of AD 2. NMN-induced memory improvement was dependent on DCT-1 function
Yao et al. (2017)	APPswe/PS1dE9 (AD-Tg)	Subcutaneous administration of NMN (100 mg/kg/day) in sterile PBS every other day for 28 days	1. NMN rescues cognitive impairments and suppresses JNK phosphorylation in AD-Tg mice 2. NMN decreases the level of Aβ and deposition and changes the processing of APP in AD-Tg mice 3. NMN improves inflammatory responses and ameliorates synaptic loss in AD-Tg mice
Wang et al. (2016)	Aβ_1__–__42_ oligomer-injected bilaterally into the lateral ventricles	Intraperitoneally administrated NMN at the dose of 500 mg/kg/day for 10 days	1. NMN restored the NAD^+^ level and prevented Aβ_1__–__42_ oligomer-induced impairment of spatial learning and memory 2. NMN decreased the Aβ_1__–__42_ oligomer-induced neuronal death of OHCs and prevented the inhibition of LTP and increased the ATP level 3. NMN decreased ROS levels in hippocampal slices exposed to Aβ_1__–__42_ oligomer and prevented the elevation in mitochondrial superoxide
Long et al. (2015)	APPswe/PS1dE9 (AD-Tg)	NMN (100 mg/kg) in sterile PBS (200 μl) subcutaneously every other day for 28 days	1. NAD^+^ reverses deficient OCR in a cell-based model of amyloid-β toxicity and as well in AD-Tg mice reversed by NMN 2. Decreased full-length mutant human APP levels in the brain of NMN-treated transgenic mice
**NR vs. placebo**			
Xie et al. (2019)	APP/PS1	NR was mixed into the mice’s food with the concentration of 2.5 g/kg for 3 months	1. NR inhibited the body weight gain of aged and APP/PS1 mice 2. NR prevented memory impairment of aged mice and improved the contextual fear memory of APP/PS1 mice 3. NR decreased the chronic neuroinflammation and Aβ accumulation in APP/PS1 mice
Hou et al. (2018)	3xTgAD and 3xTgAD/Polβ^±^	NR in their drinking water (12 mM) for 6 months	1. NR improves learning and memory in AD/Polβ mice and restores hippocampal synaptic plasticity in AD/Polβ mice 2. NR increases neurogenesis and decreases neuroinflammation and tau phosphorylation but not Aβ accumulation in AD and AD/Polβ mice 3. NR normalizes mitochondrial stress in human AD fibroblasts and decreases DNA damage and apoptosis through SIRT3 and SIRT6
Gong et al. (2013)	Tg2576 AD mouse model	Dietary treatment of NR 250 mg/kg/day for 3 months	1. NR promotes cognition coincided with induction of PGC-1α and improves synaptic plasticity in Tg2576 mice 2. NR reduces BACE1 levels through promotion of PGC-1α expression 3. NR induces PGC-1α-associated energy metabolism genes

*5xFAD mice express human APP and PSEN1 transgenes with a total of five AD-linked mutations: the Swedish (K670N/M671L), Florida (I716V), and London (V717I) mutations in APP; and the M146L and L286V mutations in PSEN1. NAM, nicotinamide; AD, Alzheimer disease; ICR, Institute of Cancer Research; NA, nicotinic acid; APP, amyloid precursor protein; PS1, presenilin 1; PARP-1, poly[ADP-ribose]polymerase 1; NF-κB, nuclear factor-κB; ROS, reactive oxygen species; MAPK, A mitogen-activated protein kinase; ERK, extracellular signal-regulated kinase; CREB, cAMP-response element binding protein; ATP, adenosine triphosphate; SIRT1, sirtuin 1; PGC-1α, peroxisome proliferator-activated receptor gamma coactivator 1-alpha; NAD, nicotinamide adenine dinucleotide; BACE1, beta-secretase 1; 3-AB, 3-aminobenzamide; OCR, oxygen consumption rate; OHCs, organotypic hippocampal slice cultures; LTP, long-term potentiation.*

### Nicotinamide vs. Placebo

In terms of NAM compared with placebo, there were seven preclinical trials in several AD models, much more than other precursors. The reason might be that NR or NMN is changed to NAM in the liver before being transformed to NAD^+^, indicating that NAM is the most direct dietary NAD^+^ precursor (Liu et al., 2018). Among them, Kim et al. (2020) intended to investigate the changes in metabolite profiles and found that NAM levels significantly decreased in 5xFAD mice at 8 or 12 months. Accordingly, treatment with NAM (10 mM) ameliorated the loss in spine density, indicating a potential role of neuroprotection. Vakilinezhad et al. (2018) adopted a sustained/controlled delivery system, called NAM-loaded SLNs to treat AD rats and showed that this brain delivery system loaded with NAM could attenuate the cognition impairment of rats and reduce the p-tau more effectively than the conventional administration of NAM in the early stage of AD. Kim and Yang (2017) reported that NAM (200 mg/kg/day) pretreatment for 7 days could significantly reduce APP and PS1 gene expression and increase the SIRT1 in brain tissues. Bayrakdar et al. (2014a,2014b) aimed to search the effects of NAM on oxidative stress, apoptosis, and the regulation of PARP-1 and NF-κB in Aβ_1__–__42_-induced neurodegeneration. NAM supplementation (either 100 or 500 mg/kg) protected against Aβ_1__–__42_-upregulated Bcl-2; reduced PARP-1, NF-κB, p53, reactive oxygen species (ROS), and Bax levels; and decreased the oxidative stress and elevated GSH levels. Moreover, Liu et al. (2013) reported that treatment with NAM (40 mg/kg/day) for 8 months resulted in improved cognitive performance, preserved mitochondrial integrity, and reduced Aβ and p-tau pathologies. Moreover, NAM augmentation elevated activities of p-Akt and ERKs and the transcription factor CREB in the hippocampus and cerebral cortex. Finally, Green et al. (2008) found that NAM reduced p-tau (Thr231) in a manner similar to the inhibition of SirT1 and also prevents against cognitive deficits in 3xTgAD mouse model.

### Nicotinamide Mononucleotide vs. Placebo

NMN is synthesized from NAM by NAMPT and NR through NRKs (Figure 1). Four studies evaluated the efficacy of NAM in the AD models. Fang et al. (2019) took advantage of the already established models of mutant tau (CK12) strain and Aβ_1__–__42_ strain (CL2355) in *C. elegans* model and found that NMN improved the memory of transgenic nematode AD models. Regarding the underlying mechanism, Fang et al. (2019) reported that NMN-induced memory improvement was dependent on DCT-1 function. Yao et al. (2017) showed that NMN (100 mg/kg) treatment dramatically decreased Aβ production, Aβ burden, synaptic loss, and inflammatory responses and that this effect depends on the inhibition of JNK activity in transgenic animals. In Aβ-injected AD-induced rats, Wang et al. (2016) found that NMN sustained improvement in cognitive function as assessed by the Morris water maze and attenuated neuronal cell death in organotypic hippocampal slice cultures (OHCs). In addition, NMN treatment also significantly prevented the Aβ oligomer-induced inhibition of long-term potentiation (LTP) and eliminated accumulation of ROS as well (Wang et al., 2016). In the end, deficiencies in oxygen consumption rate (OCR) and the levels of full-length mutant APP were successfully reversed using NMN (100 mg/kg) in a cell-based model of Aβ toxicity as well as APP/PS1 AD mice.

### NR vs. Placebo

Xie et al. (2019) found that NR inhibited the accumulation of Aβ and the migration of astrocyte to Aβ, suggesting that uptake of NR can be utilized to prevent the progression of dementia. Hou et al. (2018) reported that 3xTgAD/Polβ^±^ mice displayed a low level of cerebral NAD^+^/NADH ratio, which is normalized by NR treatment. Of note, NR treatment relieved cognitive performances in multiple behavioral tests and restored hippocampal synaptic plasticity in 3xTgAD mice and 3xTgAD/Polβ^±^ mice. Dietary treatment of NR 250 mg/kg/day for 3 months in Tg2576 AD mouse model promoted cognition function, which coincided with induction of PGC-1α and enhanced NAD^+^ expression, showing that NR might improve AD-associated memory and learning and synaptic plasticity, at least in part through facilitating PGC-1α-mediated BACE1 ubiquitination and degradation and thus preventing Aβ production in the brain (Gong et al., 2013). Only one study reported that NA 20 mg/kg/day for 7 days had no effect on the expression of APP and PS1 (Kim and Yang, 2017). Regarding other NAD^+^ precursors, such as NaR, NaMN, and NAAD, no relevant studies have ever been conducted in AD area, all of which can be considered when designing future studies.

Beside the preclinical trial of NAD^+^ precursors for AD models, several clinical trials of NAM and NADH have been conducted in AD patients. A clinical trial of NAM (1,500 mg twice daily) on 15 AD patients for 24 weeks was performed (Phelan, 2017). However, NAM did not show any positive effects for the memory performance. Apart from NAD^+^ precursors, NADH is used in clinical trial for AD patients. An open-label pilot study showed that prescription of NADH (twice a day with a total of 10 mg/day) for 8–12 weeks in 17 patients suffering from dementia of AD had a protective effect on cognitive function (Birkmayer, 1996). Further, a follow-up randomized, double-blind, clinical study with a similar design displayed a higher score of memory performance and supported NADH as a treatment for AD (Demarin et al., 2004). However, Rainer et al. (2000) reported that NADH ingestion for 3 months is unlikely to achieve cognitive improvements and present theoretical arguments against an effectiveness of this compound in dementia disorders. Based on these conflicting results, no definitive conclusion can be drawn.

## Future Perspective

The main result of this review displayed that declined NAD^+^ concentration in brain cells is common during aging, which contributes to the pathogenesis of AD. These results also support the idea that an inexpensive and safe vitamin-based intervention protects against AD pathology for neuroprotection is an attractive prospect. However, several important concerns surrounding NAD^+^ need to be addressed. First, further uncovering NAD^+^ biosynthesis, consumption, and the transport of corresponding precursors and intermediates within the cortex, hippocampus, or entorhinal cortex is essential to help in understanding NAD^+^ metabolism in these highly heterogeneous organelles. The uptake and biodistribution of NAD^+^ precursors in various tissues and cell compartment is poorly understood. Second, in view of the ubiquity of NAD^+^ metabolism in cellular health and healing, information gained in the brain may provide novel clues and translational insights that could be applicable to other organs, such as the heart. Boosting cardiac NAD^+^ contents by genetic NAMPT expression or exogenous NMN supplementation seems to prevent the cardiac damage (Hsu et al., 2009). Third, NAD^+^ precursors display efficacy in a plenty of animal disease models, prompting numerous clinical trials of NAD^+^ precursor in humans; and whether these findings will be translated to humans is the next big question. Moreover, we still have no idea which precursor is the strongest one to increase the NAD^+^ level. Hence, we should consider this point when designing future studies, and it is also essential to compare them on safety, therapeutic effects, and side effects. Moreover, there are also a couple of practical issues to consider and conquer, such as how to stabilize NAD^+^ precursors, how to best deliver them to the target and at what dose, what the best analytical methods and biomarkers are, and which the best one is to regulate NAD^+^ to achieve the expected efficacy in specific diseases.

## Conclusion

NAD^+^ is a pivotal metabolite involved in the pathophysiology of AD. This comprehensive review of emerging findings revealed key roles for NAD^+^ precursors and related metabolites in AD models and showed how NAD^+^ may affect pathological hallmarks of AD. Advances in understanding the molecular mechanisms of NAD^+^-based neuronal resilience will result in novel approaches for the treatment of AD. The data from the included studies have shown that supplementation with NAD^+^ precursors appeared to be an effective and safe anti-AD strategy with suitable bioavailability for preventing neuropathological and behavioral symptoms. Currently, the stage is set to test whether these exciting preclinical trials are precursors for success in large randomized clinical trials and whether the results can be translated into the clinic to improve AD patient phenotypes.

## Author Contributions

W-WW and LF carried out the idea and searched relevant literatures. H-JH made substantial contributions to the conception and design and figures and tables. XW, SZ, and XX drafted and revised the manuscript. C-LX and RH were involved in drafting the manuscript and supervised the process. All authors contributed to the article and approved the submitted version.

## Conflict of Interest

The authors declare that the research was conducted in the absence of any commercial or financial relationships that could be construed as a potential conflict of interest.

## Publisher’s Note

All claims expressed in this article are solely those of the authors and do not necessarily represent those of their affiliated organizations, or those of the publisher, the editors and the reviewers. Any product that may be evaluated in this article, or claim that may be made by its manufacturer, is not guaranteed or endorsed by the publisher.
